# Investigation of asymmetry reduction for surface structuring and destructuring by laser remelting

**DOI:** 10.1016/j.heliyon.2024.e24067

**Published:** 2024-01-06

**Authors:** Laura Kreinest, Benedikt Schober, Edgar Willenborg, Jochen Stollenwerk

**Affiliations:** a Fraunhofer Institute for Laser Technology, Steinbachstraße 15, 52074 Aachen, Germany; b RWTH Aachen University, Chair for Digital Additive Production, Campus-Boulevard 73, 52074 Aachen, Germany; c RWTH Aachen University, Chair for Technology of Optical Systems, Steinbachstraße 15, 50274 Aachen, Germany

**Keywords:** WaveShape, Laser material processing, Structuring, Destructuring, Shape deviation

## Abstract

Lasers are widely used for structuring metallic surfaces by ablating material. An alternative approach for laser structuring is surface structuring by laser remelting (WaveShape), which is based onthe continuous remelting of a thin surface layer using laser radiation while simultaneously modulating the laser power. The structures are generated by redistribution of the molten material. The structure height and the structure wavelength of periodic structures created using WaveShape can be precisely adjusted by the adaption of various process parameters. However, the structures produced are mostly asymmetrical. An asymmetric structure refers to a structure that is not symmetrical and is inclined in or against the scanning direction. In the context of this work, the asymmetry of the structures was significantly reduced through two different process adaptations. As a first adaption, a compensation term is added to the laser power modulation, which is calculated from the difference profile between a target profile and a structured profile. With this adaption, the shape deviation of an asymmetrical structure could be decreased by 66 %. Asymmetry can be reduced efficiently, although the difference profile required must be determined from a preliminary process step. As a second adaption, a modulation of the scanning speed is investigated with which shape deviation can be decreased by 40 %. Asymmetry is not as effectively prevented as when using the first adaption, but the adaption can be performed without the difference profile. Another aim was to investigate the destructuring, i.e. the removal and therefore smoothing, of asymmetric structures. Using the inverse laser power modulation for destructuring, the structure height of a symmetrical structure can be reduced by 91 % while the structure height of an asymmetric structure can be reduced by 68 %. To increase the efficiency of destructuring of an asymmetrical structure, iterative destructuring was investigated. With two iterations of destructuring, the structure height was reduced by 90 %. As a second approach for more efficient destructuring of asymmetric structures an adaption of the laser power modulation via a compensation term was investigated. The structure height could be reduced by 86 %. In summary, results show that asymmetry can be prevented when structuring with WaveShape and that asymmetric structures can be destructured efficiently.

## 1 Introduction

Structured surfaces are crucial in numerous industrial applications, as they play a vital role in almost every aspect of daily life. Precise non-imaging micro-optical component production, such as light guides, light pipes, light rails, retroreflectors, or lens arrays, is a highly relevant industry with a significant demand for structured surfaces. These components have wide-ranging applications in fields including automotive and architectural lighting, solar cells, Fresnel optics, and liquid crystal displays (LCDs) [[1], [2], [3]]. In addition to conventional, mechanical structuring processes, laser-based structuring processes are becoming more and more important for the structuring of metallic surfaces as they combine high precision with contactless processing. The laser-based structuring processes are divided into the widely used ablative processes and the more specific remelting processes.

In structuring processes based on remelting no material is removed but redistributed. Major laser-based structuring processes based on material redistribution by remelting are Direct Laser Interference Patterning (DLIP), Surfi-Sculpt®, Pulsed laser micro structuring (PLμs) and Structuring by Laser Remelting (WaveShape).

DLIP is a structuring method based on the interference of at least two laser beams. With the interference of the laser beams a defined intensity distribution is created which is transferred to the surface [4]. The resulting surface topography has the same geometrical dimensions as the intensity pattern [5]. The structures produced are periodic and have structure wavelengths of a few micrometers in lateral and vertical direction [6]. From the work of Aguilar-Morales et al. [7] it can be concluded that the initial roughness must be comparatively low (*Ra* = 60 nm) to create defined and distinguishable surface structures.

Another method for structuring based on remelting is Surfi-Sculpt® developed by Dance and Buxton [8]. Laser or electron radiation is focused on the sample surface, creating a melt pool and partially evaporating material. The focused beam is then moved over the surface. An elevation is created at the beginning of the track and a cavity at the end of the track. The structure height can be increased by multiple passes [9]. However, the structures produced by Surfi-Scuplt® have a large surface roughness due to melt ejection.

Pfefferkorn and Morrow [10] use PLμS for the structuring of non-periodic structures with a lateral resolution *λ* = 0.05–0.2 mm. In PLμS, the surface is melted with pulsed laser radiation. By varying the pulse duration and thus the fluence, a temperature gradient is induced in the melt pool. The resulting thermocapillary flow leads to a local redistribution of the material at the melt pool surface [10].

Wang et al. [11] investigate the nano-structuring of silicon by redistribution of material using spatially modulated femtosecond laser pulses. By shaping the laser intensity distribution, complex surface patterns conforming to the laser intensity distribution were formed. A precision of 50 nm can be achieved [12].

WaveShape is a process for structuring metal surfaces by continuously remelting a thin surface layer (<100 μm) using laser radiation while simultaneously modulating the laser power. With WaveShape, periodic structures with structure wavelengths in the micrometer to millimeter range and structure heights up to a few to several micrometers can be created [[13], [14], [15]]. Temmler et al. [13] showed that process rates of up to 17.6 mm³/min can be achieved, thus the process is faster than ablation-based structuring techniques such as USP-Ablation. Oreshkin et al. [16] found that residual surface waviness after laser polishing of roughly milled surfaces can be destructured by means of a variant of WaveShape called “Active Wave Reduction (AWR)”. With AWR, the inverse structure of the structure already present on the surface is created and the surface is thus smoothed by destructive superposition. Kreinest et al. [17] investigated the destructuring of residual waviness on additive manufactured parts and showed that a reduction of 85 % is feasible. Therefore, WaveShape could also be an interesting post-processing alternative for additive manufactured components.

The structures created by WaveShape are mostly characterized by their profile height and structure wavelength. While the structure wavelength corresponds to the modulation wavelength of the laser power modulation, Temmler et al. [13,18,19] found that the profile height of the structures can be adapted via a large number of process parameters.

Surface structures created with WaveShape generally correspond to the laser power modulation, resulting in precise structuring. However, structures created by WaveShape are asymmetrical within a wide range of process parameters [20] which reduces the precision of the structuring. An asymmetric structure refers to a structure that is not symmetrical and is inclined in or against the scanning direction. Temmler and Pirch [21,22] show with the numerical results based on an FEM model that the different dynamics of the melting and solidification front as well as the vapor pressure that occurs when the boiling temperature is reached are reasons for the asymmetry of the structures. When the laser power amplitude and thus the energy input into the melt pool is large enough for the molten material to reach the boiling temperature, structures become asymmetrical although a symmetrical, sinusoidal laser power modulation is used for structuring. Oreshkin et al. [23] demonstrate an approach to reduce the asymmetry of sinusoidal structures using a compensation term with half the structure wavelength which is added to the calculation of the laser power modulation.

A main factor limiting the structure accuracy and the precision of the WaveShape process is the asymmetry of the structures. One aim of this work is therefore to increasing the accuracy of sinusoidal structures obtained with WaveShape. Oreshkin et al. [23] show that a reduction of the asymmetry is possible by an adaptation of the laser power modulation. The disadvantage of this adaptation is, however, that an extensive process parameter variation of the phase shift and the laser power amplitude must be carried out beforehand. Therefore, an adaptation is to be developed with which the asymmetry can be reduced without having to carry out a time-consuming process parameter study. The aim is to adapt the laser power modulation in such a way that the asymmetry of the structures is reduced during structure formation. For this purpose, two adaptations of the laser power modulation are investigated. As a first adaption a compensation term is added to the laser power modulation, which is calculated from the difference profile between a target profile and a structured profile. This adaption is similar to the one of Oreshkin et al. [23], but instead of an extensive parameter study, only one test field needs to be structured in advance. As a second adaption, a modulation of the scanning speed is investigated.

Another aim is to investigate the destructuring of asymmetric structures. Destructuring and WaveShape are basically the same process. The essential difference is that WaveShape is carried out on flat surfaces and the destructuring on surfaces on which a structure is already present. While Oreshkin et al. [16] showed that symmetric structures can be reduced, the extent to which asymmetrical structures can be destructured has not yet been investigated. To efficiently destructure asymmetrical structures, it is analyzed to what extent on the one hand iterative destructuring and on the other hand destructuring with an adaption of the laser power modulation via a compensation term can reduce the structure height.

## 2 Materials and methods

### 2.1 Process principle and process parameters

**Fig. 1:**
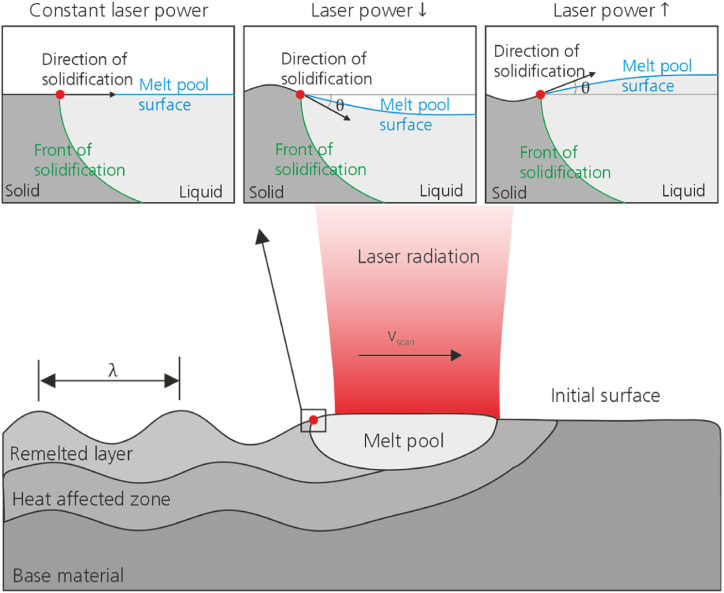
Schematic representation of the structure formation during WaveShape, according to Temmler [20].

The structure formation during WaveShape is shown schematically in Fig. 1. Temmler and Pirch [21,22] describe that during the formation of the melt pool, the density discontinuity between solid and liquid phase resulting from the phase transition as well as the thermal expansion of the melt lead to deformation of the melt pool surface. During remelting with constant laser power, the surface of the melt pool is approximately flat in the area of the solidification front and the surface is not structured. Structuring occurs when the laser power is either increased or decreased during the remelting process, resulting in an increase or decrease of the melt pool volume. The curvature of the melt pool is continuously changed by the modulation of the laser power and the three-phase line is moved out of the x,y-plane in positive or negative z-direction. A convex or concave curvature of the melt pool surface thus leads to a change in the direction of the solidification front along the three-phase line. This changes the direction of solidification, leading to the formation of the structures.

To create a sinusoidal structure, the laser power *P*_*L*_ is modulated sinusoidally around the average laser power *P*_*M*_ with the laser power amplitude *P*_*A*_ and the structure wavelength *λ* according to

$$P_{L}\left(x\right)=P_{M}+P_{A}∙\sin\left(\frac{2\pi }{\lambda }x\right)\text{.}$$

A unidirectional scanning strategy is typically used for WaveShape. The laser beam with the laser beam diameter *d*_*L*_ is moved over the surface of the sample with a constant scanning velocity *v*_*scan*_, a track offset *dy* and *n* number of passes. The process parameter ranges employed for Waveshape and destructuring in this study are displayed in Table 1 and Table 2.

**Table 1:** Process parameters used for WaveShape.

Process parameter	Average laser power P_M_ [W]	Laser power amplitude P_A_ [W]	Structure wavelength λ [mm]	Beam diameter d_L_ [μm]	Scanning velocity v_scan_ [mm/s]	Track offset dy [μm]	Number of passes n
Range	119	40.5	1; 2	250	5–50	62.5	1

**Table 2:** Process parameters used for destructuring.

Process parameter	Average laser power P_M_ [W]	Laser power amplitude for structuring P_A,A_ [W]	Laser power amplitude for destructuring P_A,B_ [W]	Structure wavelength λ [mm]	Beam diameter d_L_ [μm]	Scanning velocity v_scan_ [mm/s]	Track offset dy [μm]	Number of passes n
Range	119	40.5	24.3–48.6	1; 2	250	5–50	62.5	1–2

### 2.2 Opto-mechanical set-up

**Fig. 2:**
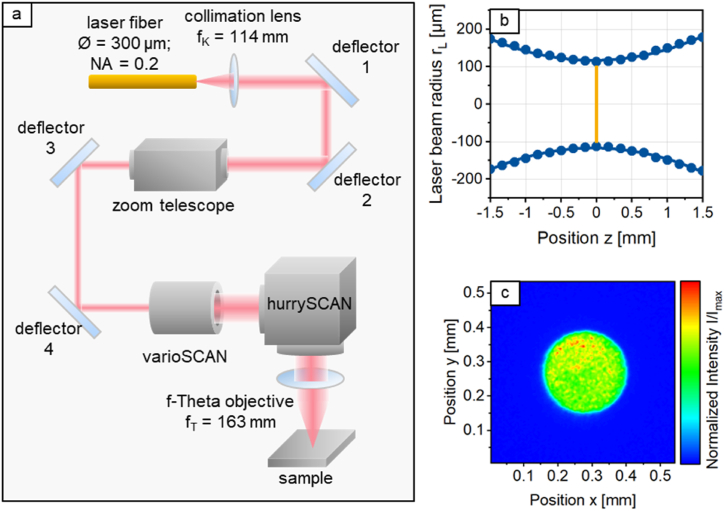
a: Schematic of the laser beam path and crucial optical elements. b: Caustic of the laser beam; c: 2D-intensity distribution of the laser beam in the focal plane (marked by the orange line) with a laser beam diameter of d_L_ = 250 μm. (For interpretation of the references to colour in this figure legend, the reader is referred to the Web version of this article.)

All experiments were conducted with the opto-mechanical set-up schematically shown in Fig. 2. The laser source used in this set-up is a redPOWER fiber laser (SPI Laser Ltd.) emitting at a wavelength of *λ*_*em*_ = 1080 nm with maximal laser power of 500 W. The optical elements for guiding and forming the laser beam are a fiber with a diameter of *Ø* = 300 μm, a collimation lens with a focal length of *f*_*k*_ = 114 mm, a f-theta objective with a focal length of *f*_*T*_ = 163 mm and a zoom telescope with which the laser beam diameter in the focal plane can be adjusted continuously in the range of *d*_*L*_ = 120–625 μm. A 3D scanning system consisting of a hurrySCAN30 and a varioSCAN 40 (Scanlab GmbH) was used to move the focused laser beam with a velocity of up to 3 m/s over the sample surface. The hurrySCAN 30 provides movement in the x- and y-plane, while the varioSCAN 40 provides additional movement of the laser beam focus in the z-direction. The workpiece is positioned for machining with the aid of the mechanical axes. The opto-mechanical set-up is attached to a Z-axis, which allows the laser beam focus to be positioned relative to the workpiece. A process chamber with Argon atmosphere was used during processing to avoid oxidation. The residual oxygen content was <100 ppm and monitored with an oxygen meter SGM5T (Zirox GmbH). The X- and Y-axes located beneath the process gas chamber are utilised for the lateral positioning of the workpiece to the laser beam focus. The zero position of the scanner was positioned in the center of the respective test field for all experiments. All axes are controlled via an NC control.

The caustic of the laser beam was characterized with a MicroSpotMonitor “MSM” (Primes GmbH). Fig. 2b and c shows the caustic of the laser beam in and close to the focal plane and the size and intensity distribution in the focal plane. The laser beam diameter in the focal plane is approx. *d*_*L*_ = 250 μm (by 86 % energy inclusion) with a *M*^*2*^ = 30.4. The Rayleigh length is calculated to *r*_*R*_ = 1.51 mm and the divergence angle to *ϴ* = 144.4 mrad. The intensity distribution is close to an ideally shaped top-hat intensity distribution.

### 2.3 Materials and sample preparation

The experiments were conducted on samples of tool steel AISI H11 (1.2343, X37CrMoV5-1). The material was processed with electroslag remelting (ESR) and has a high homogeneity, purity and a fine grain structure. The chemical composition according to the manufacturer “Deutsche Edelstahlwerke (DEW)” is given in Table 3.

**Table 3:** Chemical composition of AISI H11 from the data sheet of the manufacturer.

Element	C	Si	Mn	Cr	Mo	V	Fe
Fraction [wt.-%]	0.37	1.00	0.40	5.30	1.30	0.40	Bal.

To produce samples, rectangles with a thickness of 10 ± 0.04 mm were cut from rectangular bar material with an edge length of 100 ± 0.1 mm so that all samples came from the same batch of material. The discs were then milled on both sides to achieve plane parallelism of approx. 25 μm. Afterwards, all samples were remelted with laser radiation (melt depth of approx. 50 μm) to homogenize the samples and evaporate any non-metallic inclusions in the remelted zone. Before processing, all samples were cleaned with isopropanol.

### 2.4 Measurement of surface topography

The surface topography is measured with a Nexview NX2 (Zygo Corporations) using white light interferometry (WLI). The resolution of the surface measurements in lateral direction is 3.14 μm and <1 nm in vertical direction. Several individual images are stitched together with an overlap of 20 %. The profile height of the generated structures is in the micrometer range. The measurement error is thus at least one order of magnitude smaller than the measured values and can be neglected. For the evaluation of the generated and destructured structures, an F-operator is applied to the measured surface topography data. For this purpose, the measured data are masked and divided into a reference and test surface. The unprocessed initial state is defined as the reference surface. An F-operator with a 4th order polynomial is applied to the reference surface. The resulting compensation surface is then subtracted from the total surface topography.

### 2.5 Calculation of shape deviation

**Fig. 3:**
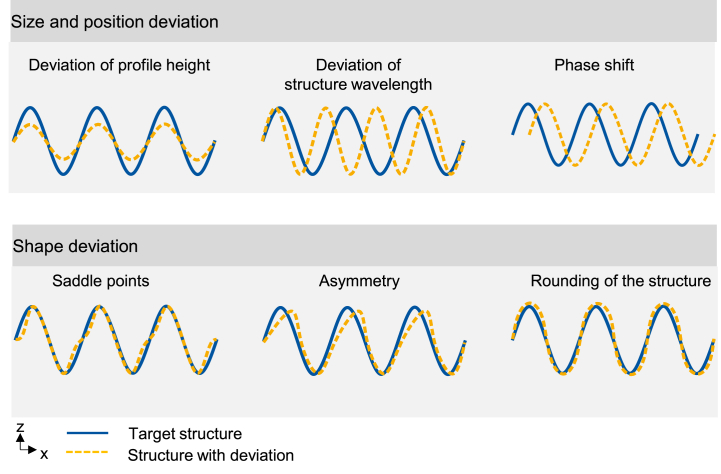
Schematic representation of the structure deviation subdivided into size and position deviation as well as shape deviation.

Various types of structure deviations are described by Oreshkin et al. [24], which can basically be divided into the two categories “size and position deviation” and “shape deviation”. Fig. 3 schematically shows examples from both categories. The evaluation of the structures created with WaveShape is mostly based on size and position deviations like deviations of the structure wavelength, profile height, profile gradient and phase shift between laser power modulation and created structure. Nevertheless, shape deviations and foremost the asymmetry are also decisive to be able to assess whether the desired target structure is reproduced on the sample surface with sufficient structural accuracy. For a quantitative analysis of the asymmetry of the structures, a measurement method for determining the shape deviation was developed.

With this method the shape deviation *Pa(Δ)* is calculated as the quantitative deviation of the created structure *h*_*Created*_*(x)* from the target structure *h*_*Target*_*(x)*. The shape deviation is calculated as the mean arithmetic height of the difference structure *Δ(x)* between the created and the target structure according to

$$Pa\left(\Delta \right)=\min\left(\frac{1}{L}\sum_{x=0}^{x=L}\left|\Delta \left(x\right)-\bar{\Delta \left(x\right)}\right|\right)\;with$$

$$\Delta \left(x\right)=\|h_{Created}\left(x\right)\left\|\cdot S-{\|h}_{Target}\left(x-x_{0}\right)\right\|\text{.}$$

For a profile height and phase shift independent calculation of the shape deviation, the actual profile and the target profile are normalized via the PV-value (peak-to-valley value) to values between [−1; 1]. Since the PV-value can be influenced by both, local roughness peaks and measurement artefacts, a factor for scaling the height *S* of the created profile is considered when calculating the difference profile. In addition, a shift *x*_*0*_ of the created profile in x-direction is considered to calculate the difference profile independently of the phase shift.

The calculation of the shape deviation presents an extreme value problem with the two variables *S* and *x*_*0*_. By a computer-aided variation of the two variables, the combination of scaling and phase shift are determined at which the shape deviation becomes minimal. In this work, the scaling is varied with an increment of 0.01 and the phase shift in the x-direction with an increment of 0.05 mm.

The smaller the determined shape deviation, the higher is the match between the target profile and the created profile. At a value of *Pa(Δ)* = 0, the target and created profiles match exactly, while at a value of *Pa(Δ)* = 0.5, the created and target profiles differ by 50 %.

The calculated shape deviation increases with the selected measuring length L. The reason is that with longer measuring lengths, the shape deviation at longer structure wavelengths is also considered in the calculation. For the calculation of the shape deviation of periodic structures, a measuring length of *L* = 5*λ is specified following the calculation of roughness according to DIN EN ISO 25178-2.

## 3 Results and discussion

### 3.1 Structuring

#### 3.1.1 Shape deviation of symmetrical and asymmetrical structures

Since the determination of the shape deviation is a new method, no reference values for symmetrical and asymmetrical structures exist so far. Therefore, the shape deviation of a symmetrical and an asymmetrical structure is determined. The aim is to classify the magnitude of *Pa(Δ)*, i.e. to determine how large *Pa(Δ)* is for an asymmetrical structure as well as for a symmetrical structure.

The extent of the asymmetry depends on the structure wavelength. At small structure wavelengths (*λ* < 4*d_*L*_), the structures are usually inclined against the scanning direction. For large structure wavelengths (*λ* > 4*d_*L*_), an inclination in the scanning direction is observed. Symmetrical structures are observed in the structure wavelength range where the largest profile height is obtained (*λ* = 4*d_*L*_) [20].

**Fig. 4:**
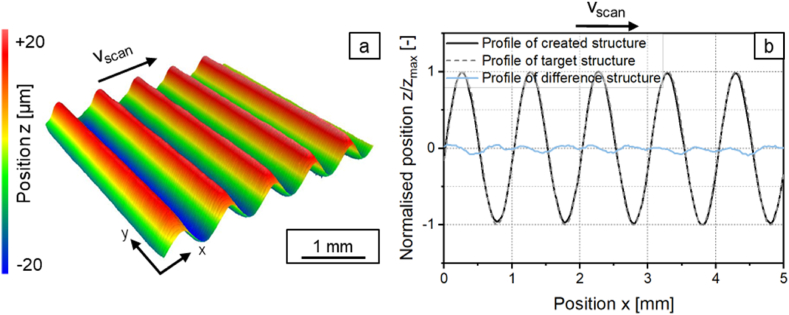
a: Surface topography and b: Measured profile and difference profile for a structure created with a sinusoidal laser power modulation with *λ* = 1 mm with *d*_*L*_ = 250 μm; *P*_*M*_ = 119 W; *P*_*A*_ = 40.5 W; *v*_*scan*_ = 50 mm/s; *dy* = 62.5 μm; *n* = 1.

**Fig. 5:**
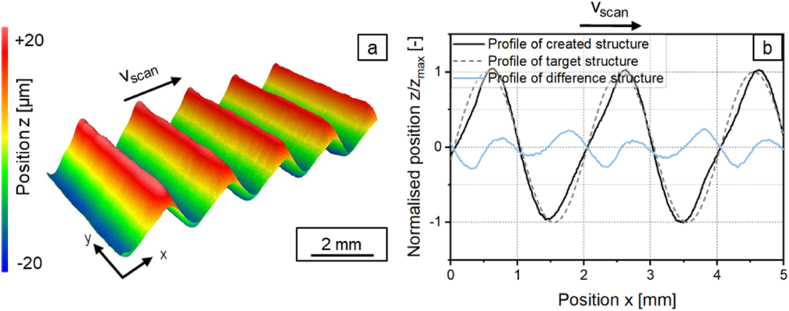
a: Surface topography and b: Measured profile and difference profile for a structure created with a sinusoidal laser power modulation with *λ* = 2 mm with *d*_*L*_ = 250 μm; *P*_*M*_ = 119 W; *P*_*A*_ = 40.5 W; *v*_*scan*_ = 50 mm/s; *dy* = 62.5 μm; *n* = 1.

To compare the shape deviation of a symmetric structure and an asymmetric structure, structures with a structure wavelength of *λ* = 1 mm and *λ* = 2 mm were structured with a sinusoidal laser power modulation and with a laser power amplitude of *P*_*A*_ = 40.5 W. With the beam diameter of *d*_*L*_ = 250 μm, this corresponds to the structural wavelength ranges in which symmetrical (*λ* = 1 mm) and asymmetrical (*λ* = 2 mm) structures are expected. The surface topography of the generated structures are shown in Fig. 4, Fig. 5a and the profiles of the generated and targeted structures and their respective differences are shown in Fig. 4, Fig. 5b.

The created structure with *λ* = 1 mm is symmetrical while the generated structure with *λ* = 2 mm is asymmetrical. The shape deviation for a symmetrical structure with a structure wavelength of *λ* = 1 mm is *Pa(Δ)* = 0.043 and for an asymmetrical structure with a structure wavelength of *λ* = 2 mm is *Pa(Δ)* = 0.122. The asymmetry of a structure can thus now be evaluated quantitatively with the method described in chapter 2.5.

#### 3.1.2 Asymmetry reduction using a compensation term

To prevent asymmetrical structure formation, two approaches were investigated. One approach is to reduce the asymmetry by adding a compensation term to the laser power modulation. In contrast to the method of Oreshkin et al. [23], the compensation term applied represents the calculated difference profile between the symmetrical target structure and the created asymmetrical structure. From the target structure *h*_*Target*_ and the difference structure *Δ(x)*, a modified structure *h*_*Mod*_ is calculated according to:

$$h_{\mathrm{Mod}}\left(x\right)=h_{Target}\left(x\right)-A_{\Delta }∙\Delta \left(x\right)\text{.}$$

where *A*_*Δ*_ represents a factor for the weighting of the difference profile. The calculation of the laser power modulation *P*_*L*_*(x)* is then calculated with the integral approach formulated by Kreinest et al. [17] in accordance with

$$P_{L}\left(x\right)=P_{M}+P_{A}∙\left\|\int_{0}^{L}h_{\mathrm{Mod}}\left(x\right)∙dx\right\|$$

with the average laser power *P*_*M*_, the laser power amplitude *P*_*A*_ and the profile length *L*. The numerical calculations by Temmler and Pirch [21,22] show that a change of laser power induces a change of melt pool volume. This leads to a change in melt pool geometry and thus to the structure formation. The shape and geometry of the melt pool surface is therefore determined by the change of laser power. Kreinest et al. [17] assume that a height profile generated by means of WaveShape corresponds to the derivative of the laser power. Therefore, in the formulated approach, the laser power modulation is calculated by integrating the target profile.

**Fig. 6:**
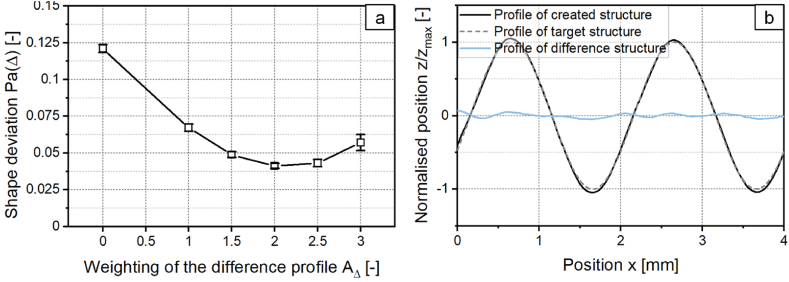
a: Shape deviation for a structure created with a sinusoidal laser power modulation adding a compensation term. b: Profile of target structure, created structure and difference structure for *A*_*Δ*_ = 2 with *λ* = 2 mm; *d*_*L*_ = 250 μm; *P*_*M*_ = 119 W; *P*_*A*_ = 40.5 W; *v*_*scan*_ = 50 mm/s; *dy* = 62.5 μm; *n* = 1.

In the following it is investigated whether the modification of the target structure by a compensation term leads to a reduction of asymmetry. For this purpose, the modified target structure of the asymmetric structure with a structure wavelength of *λ* = 2 mm and the related laser power modulation are calculated. Subsequently, structures are generated with the calculated laser power modulations while *A*_*Δ*_ is varied to examine how the difference profile must be weighted to achieve the highest reduction of asymmetry. The shape deviation of the structures which are created with the compensation is shown in Fig. 6a as a function of *A*_*Δ*_. For *A*_*Δ*_ = 0 no compensation term was added.

With an increase of the weighting of the difference profile up to *A*_*Δ*_ = 2, the shape deviation is reduced up to 66 % and decreases from *Pa(Δ)* = 0.121 at *A*_*Δ*_ = 0 to *Pa(Δ)* = 0.041 at *A*_*Δ*_ = 2. With a further increase of *A*_*Δ*_, the shape deviation increases again. Fig. 6b shows the profiles of the structure created with a compensation term with the weighting *A*_*Δ*_ = 2, the sinusoidal target structure and the difference structure. The shape deviation obtained when structuring a sinusoidal structure with *λ* = 2 mm using a compensation term is comparable to the shape deviation of the symmetrical structure created with *λ* = 1 mm. Therefore, asymmetric structure formation can be prevented in this case when using a laser power modulation with a compensation term.

With this adaption asymmetry can be reduced without having to perform a time-consuming process parameter study. However, the disadvantage of the described method is that the difference profile of the target structure and the structure created in a first process step without adjustment, is required. Thus, the creation of the structure with adaptation always must take place on a second test field. For a industrial use, this would lead to more material usage, longer processing times and thus higher costs. Therefore, an alternative method to reduce the asymmetry in one process step is investigated.

#### 3.1.3 Asymmetry reduction by adapting the scanning velocity

To adjust asymmetry, a modulation of the scanning velocity during the process is investigated. Temmler and Pirch [21,22] found that the asymmetry of the structures, among other reasons, is due to different dynamics of the melt and solidification front. In an FEM-simulation for the creation of a sinusoidal structure on H11 steel with *v*_*scan*_ = 50 mm/s, *λ* = 1 mm, *P*_*M*_ = 115 W and *P*_*A*_ = 50 W, the maximum velocity of the melt pool front was determined to be 5.5 mm/s and the maximum velocity of the solidification front to be 8 mm/s relative to the scanning velocity. The difference in velocity between the melt and solidification front is thus up to 13.5 mm/s. The maximum difference in velocity between the melt and the solidification front occurs when the laser power decreases during the sinusoidal laser power modulation [21,22].

**Fig. 7:**
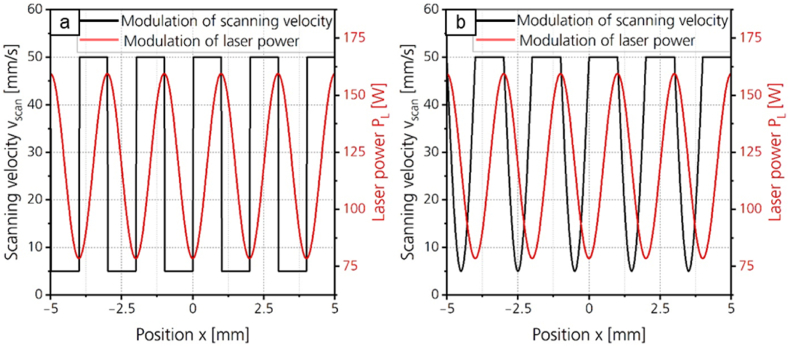
Adaptation of the scanning velocity to reduce asymmetry during WaveShape. a: Reduced scanning velocity is constant. b: Scanning velocity is reduced and increased sinusoidally.

This approach to reduce asymmetry is based on the idea to keep the velocity of the solidification front, at which the structure formation takes place, constant. Therefore, the scanning speed is reduced whenever the slope of the laser power modulation curve is negative. Two different adaptations of the scanning speed are investigated. In the first adaptation, shown in Fig. 7a, the value of the reduced scanning velocity is constant. In the second adaptation, shown in Fig. 7b, the scanning velocity is reduced and increased sinusoidally. In contrast to the first adaptation, this results in a continuous acceleration.

**Fig. 8:**
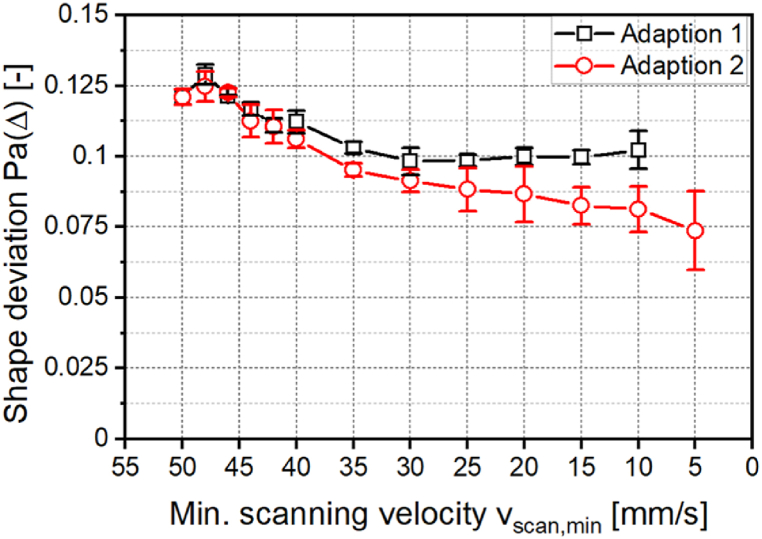
Shape deviation for structures created with a sinusoidal laser power modulation and adaption of the minimal scanning velocity with *λ* = 2 mm; *d*_*L*_ = 250 μm; *P*_*M*_ = 119 W; *P*_*A*_ = 40.5 W; *dy* = 62.5 μm; *n* = 1.

Fig. 8 shows the shape deviation as a function of the minimum scanning velocity *v*_*scan,min*_ for both adaptations. The minimum scanning velocity is introduced for the comparability of both adaptations and describes the respective smallest scanning velocity during the laser power modulation.

The first data point of both curves at a minimal scanning velocity of *v*_*scan,min*_ = 50 mm/s represents the structuring without adaptation. At a minimum scanning velocity of *v*_*scan,min*_ = 48 mm/s, the shape deviation increases by 0.008 for adaption 1 and by 0.003 for adaption 2. For a further reduction of the minimal scanning velocity, the shape deviation decreases for both adaptations. For adaption 1, the shape deviation decreases to *Pa(Δ)* = 0.098 with a minimal scanning velocity of *v*_*scan,min*_ = 30 mm/s. A further reduction of the minimal scanning velocity leads to no further reduction of shape deviation outside the standard deviation. For adaption 2, the shape deviation decreases to *Pa(Δ)* = 0.074 with a minimal scanning velocity of *v*_*scan,min*_ = 5 mm/s. Adaption 2 thus leads to a higher reduction of shape deviation. The shape deviation for a sinusoidal structure with *λ* = 2 mm and adaption of the scanning velocity is 72 % higher than the shape deviation of the symmetrical structure created with *λ* = 1 mm.

When modulating the scanning velocity, asymmetric structure formation is not prevented as effectively as with laser power modulation with a compensation term. However, the adaption can be performed without a process parameter study and the difference structure is not required.

### 3.2 Destructuring

**Fig. 9:**
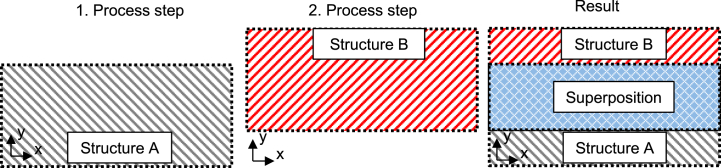
Schematic illustration of destructuring.

The procedure for investigating the destructuring process is shown schematically in Fig. 9. In a first process step, a structure A is created. In a second process step, a structure B is created, which is intended to destructure structure A by “destructive superposition”. The result is a test field from which the first, the second and the superposition of both structures can be determined.

The laser power modulation for creating structure B is initially calculated as the inverse laser power modulation of the laser power modulation used for creating structure A. The laser power amplitude for generating structure A will be referred to as *P*_*A,A*_ and the laser power amplitude for generating structure B as *P*_*A,B*_. The aim is to investigate how far the structure height of structure A can be reduced through destructuring. The structure height of the primary profile is calculated as the mean arithmetic height *Pa* for the profiles extracted from the measured surface topography. A mean value and the corresponding standard deviation are calculated from the three calculated mean arithmetic heights.

#### 3.2.1 Destructuring of symmetrical and asymmetrical structures

**Fig. 10:**
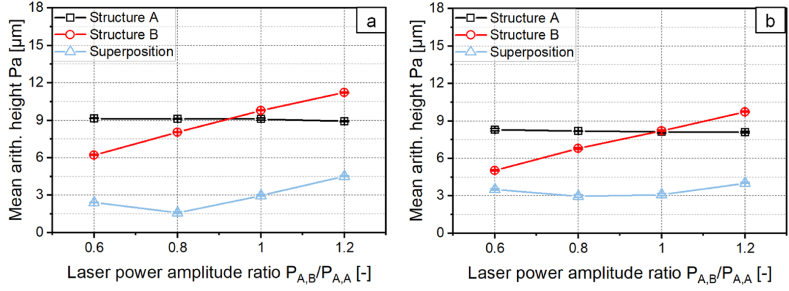
Mean arithmetic height of structure A, Structure B and superposition for a: symmetric structure with *λ* = 1 mm. b: asymmetrical structure with *λ* = 2 mm with *v*_*scan*_ = 50 mm/s; *d*_*L*_ = 250 μm; *P*_*M*_ = 119 W; *P*_*A,A*_ = 40.5 W; *dy* = 62.5 μm.

In the following chapter the symmetrical and asymmetrical sinusoidal structures shown in Fig. 4, Fig. 5 are destructured. Fig. 10a shows the mean arithmetic height as a function of the ratio of the laser power amplitudes *P*_*A,B*_*/P*_*A,A*_ for the symmetrical structure with *λ* = 1 mm. Fig. 10b shows the same for the asymmetrical structure with *λ* = 2 mm. The laser power amplitude *P*_*A,B*_ used for creating structure B was systematically varied and the laser power amplitude *P*_*A,A*_ used for creating structure A was kept constant. As expected, the mean arithmetic height of structure A is for both cases constant within the standard deviation as the laser power amplitude *P*_*A,A*_ was not varied. The mean arithmetic height of structure B increases for both cases linearly with the increase of *P*_*A,B*_.

**Fig. 11:**
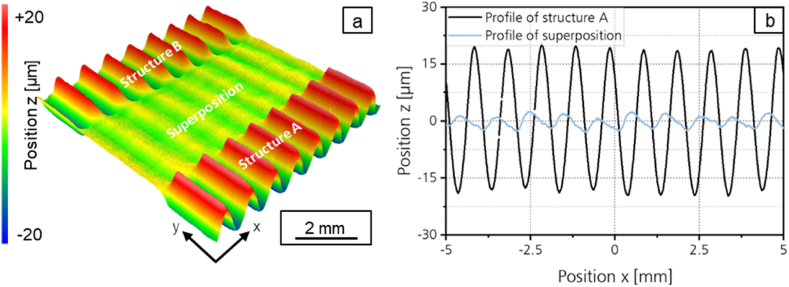
a: Surface topography and b: Profile of the symmetrical structure with *λ* = 1 mm; *P*_*A,B*_*/P*_*A,A*_ = 0.8; *v*_*scan*_ = 50 mm/s; *d*_*L*_ = 250 μm; *P*_*M*_ = 119 W; *P*_*A,A*_ = 40.5 W; *dy* = 62.5 μm.

The mean arithmetic height of the superposed structures is smaller than that of structure A or structure B for all investigated laser power amplitudes *P*_*A,B*_. For the destructuring of the symmetrical structure, the minimum of the mean arithmetic height is reached with *Pa* = 1.60 μm with a laser power amplitude ratio of *P*_*A,B*_*/P*_*A,A*_ = 0.8. The structure height of structure 1 could thus be reduced by 83 % through destructuring. Fig. 11a shows the surface topography of the symmetrical structure A and Fig. 11b the profile and the superposed structure for *P*_*A,B*_*/P*_*A,A*_ = 0.8.

**Fig. 12:**
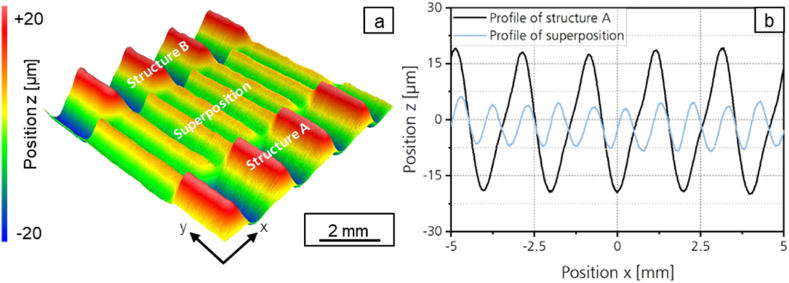
a: Surface topography and b: Profile of the asymmetric structure with *λ* = 2 mm; *P*_*A,B*_*/P*_*A,A*_ = 1; *v*_*scan*_ = 50 mm/s; *d*_*L*_ = 250 μm; *P*_*M*_ = 119 W; *P*_*A,A*_ = 40.5 W; *dy* = 62.5 μm.

The minimum of the mean arithmetic height for destructuring the asymmetric structure is reached with *Pa* = 3.0 μm at a laser power amplitude ratio of *P*_*A,B*_*/P*_*A,A*_ = 0.8 and *P*_*A,B*_*/P*_*A,A*_ = 1. The structure height of structure A could be reduced by 64 %. Fig. 12a shows the surface topography and Fig. 12b the profile of the asymmetrical structure A and the superposed structure for *P*_*A,B*_*/P*_*A,A*_ = 1.

**Fig. 13:**
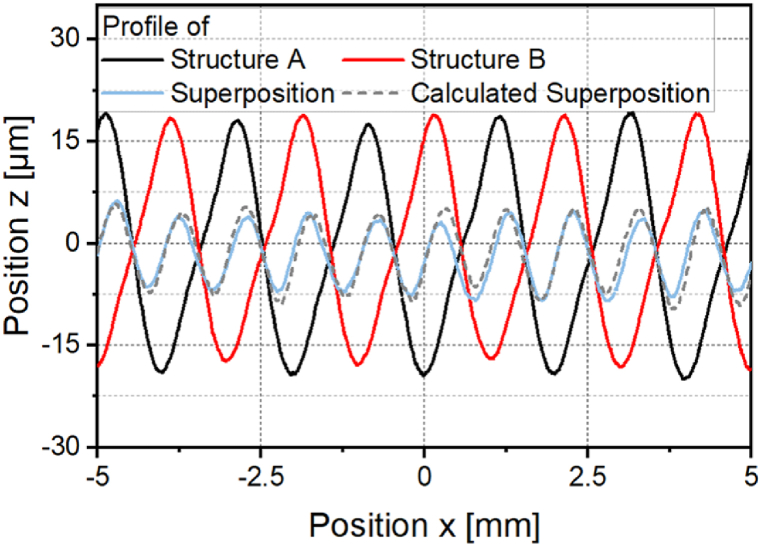
Profile cross section of Structure A, Structure B, measured and calculated superposition with *P*_*A,B*_*/P*_*A,A*_ = 1; *v*_*scan*_ = 50 mm/s; *d*_*L*_ = 250 μm; *P*_*M*_ = 119 W; *P*_*A,A*_ = 40.5 W; *dy* = 62.5 μm.

With the method presented, the mean arithmetic height of the asymmetrical structure cannot be destructured as effectively as that of the symmetrical structure. The reason for this can be derived from an examination of the profile of the asymmetrical structure A, structure B and their superposition shown in Fig. 13. Structure A and structure B are both inclined in the scanning direction. The absolute value of the positive slope is therefore smaller than the absolute value of the negative slope of the profiles. Since both structures show the same asymmetry, structure B is not the inverse structure of structure A. Therefore, the calculated superposition of both structures does not result in a smoothed profile but in a sinusoidal structure which is very similar to the measured superposition. The calculated structure shows a structure wavelength of *λ* = 1 mm, which corresponds to half the structural wavelength of structures A and B.

#### 3.2.2 Iterative destructuring of asymmetrical structures

**Fig. 14:**
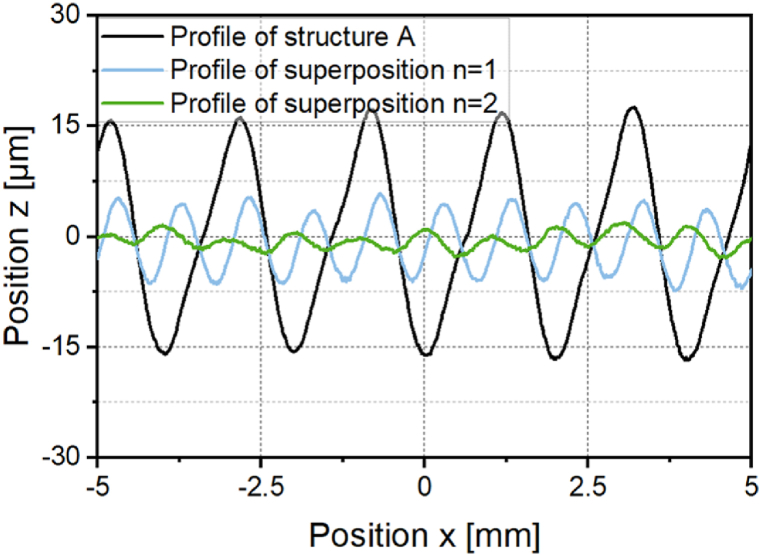
Profiles of the asymmetrical structure A and superpositions after iterative destructuring with *P*_*A,B*_*/P*_*A,A*_ = 1 (n = 1); *P*_*A,B*_*/P*_*A,A*_ = 0.17 (*n* = 2); *v*_*scan*_ = 50 mm/s; *d*_*L*_ = 250 μm; *P*_*M*_ = 119 W; *P*_*A,A*_ = 40.5 W; *dy* = 62.5 μm.

**Fig. 15:**
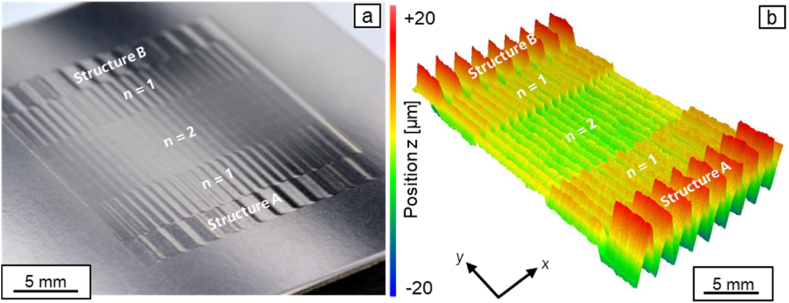
a: Photography and b: surface topography of structure A, structure B and the superimposed structures after one and after two destructuring steps.

In the following, further approaches are explored to smooth the asymmetrical structure more effectively. As a first approach, iterative destructuring is investigated. As shown in chapter 3.2.1, symmetrical structures can be destructured effectively. Therefore, the superposed structure created after a first destructuring of the asymmetrical structure is destructured again in a second process step. Fig. 14 shows the profile of the asymmetric structure A and the superimposed structures after the first destructuring (*n* = 1) and after the second destructuring (*n* = 2). For the second destructuring, a laser power modulation with a structure wavelength of *λ* = 1 mm was generated and a laser power amplitude ratio of *P*_*A,B*_*/P*_*A,A*_ = 0.17 was used. Fig. 15a shows a photography and Fig. 15b a surface topography image of structure A, structure B and the superposed structures after one and after two destructuring steps.

After the second destructuring step, a mean arithmetic height of *Pa* = 0.967 is determined for the superposition. The mean arithmetic height could be reduced by 90 % in two iterations. The reduction achieved is thus even larger than the reduction achieved when destructuring the symmetrical structure.

#### 3.2.3 Destructuring of asymmetric structures using a compensation term

**Fig. 16:**
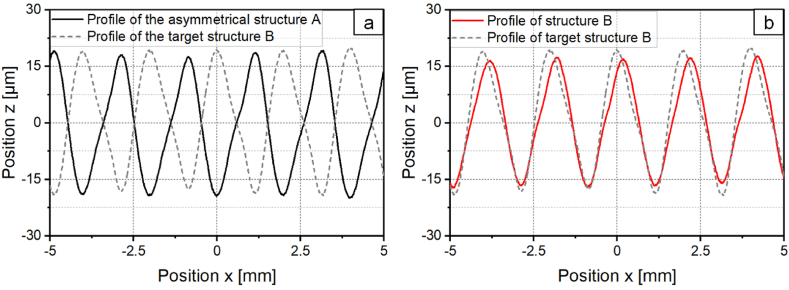
Profiles of the a: asymmetrical structure A and target structure B b: target structure B and created structure B with *P*_*A,B*_ = 40.5; *v*_*scan*_ = 50 mm/s; *d*_*L*_ = 250 μm; *P*_*M*_ = 119 W; *dy* = 62.5 μm.

The analysis of Fig. 13 shows that structuring with the inverse laser power modulation does not create the inverse structure of the asymmetric structure A. For more effective destructuring of the asymmetric structure, the inverse structure of structure A must be created as a structure B. Fig. 16a shows the asymmetric structure A and the inverse target structure to be created as structure B. One approach for creating the inverse target structure h_Target_ is to use the integral approach developed by Kreinest et al. [17] where the laser power modulation is calculated according to

$$P_{L}\left(x\right)=P_{M}+P_{A}∙\left\|\int_{0}^{L}h_{Target}\left(x\right)∙dx\right\|$$

with the average laser power *P*_*M*_, the laser power amplitude *P*_*A*_ und the structure length *L*. Fig. 16b shows a profile of the structure created with the integral approach compared to the inverse target structure. For structuring a laser power amplitude of *P*_*A,A*_ = 40.5 W was used. A shape deviation of *Pa(Δ)* = 0.154 was determined between the target structure and the actual structure. The inverse target structure cannot be generated with sufficient structural accuracy for the destructuring using the integration approach.

**Fig. 17:**
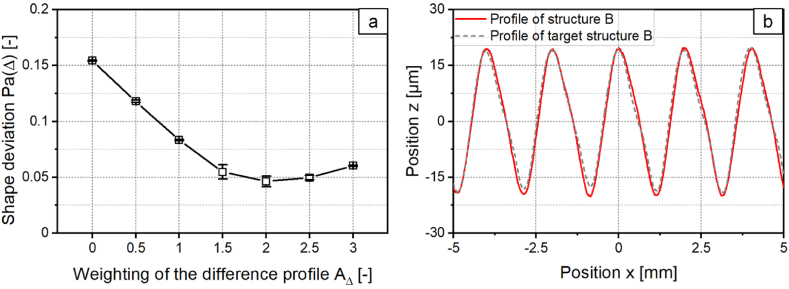
a: Shape deviation for structure B created with a laser power modulation with compensation term. b: Profiles of the target structure B and created structure B with *P*_*A,B*_*/P*_*A,A*_ = 0.8; *v*_*scan*_ = 50 mm/s; *d*_*L*_ = 250 μm; *P*_*M*_ = 119 W; *P*_*A,A*_ = 40.5 W; *dy* = 62.5 μm.

To reduce the shape deviation, a compensation term is added to the laser power modulation. As described in chapter 3.1.2, the compensation term is calculated from the difference structure of the target and the created structure. The shape deviation of the structures created with the compensation term is shown in Fig. 17a as a function of *A*_*Δ*_. The shape deviation is, similar as in Fig. 6a, reduced with increasing *A*_*Δ*_ and decreases up to 70 % at *A*_*Δ*_ = 2 to *Pa(Δ)* = 0.046. Fig. 17b shows the corresponding profile cross-sections of the target structure and the created structure with *A*_*Δ*_ = 2.

**Fig. 18:**
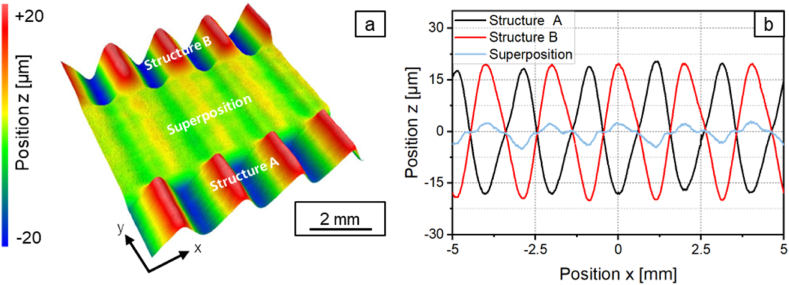
a: Surface topography and b: Profiles of asymmetrical structure A and structure B created with a compensation term and superposition with *P*_*A,B*_*/P*_*A,A*_ = 0.8; *v*_*scan*_ = 50 mm/s; *d*_*L*_ = 250 μm; *P*_*M*_ = 119 W; *P*_*A,A*_ = 40.5 W; *dy* = 62.5 μm.

By structuring with the compensation term, the inverse target structure from Fig. 16a can be structured with high structural accuracy. In a next step, the asymmetric structure A is destructured with the created inverse structure B. Fig. 18a shows the surface topography and Fig. 18b the profiles of both structures and of the superposition. The mean arithmetic height of structure A could be reduced by 86 % to *Pa* = 1.48 μm. The reduction of the mean arithmetic height is thus larger than for destructuring with inverse laser power modulation, but smaller in comparison to iterative destructuring.

## 4 Conclusion

In this study, it was shown that the structural accuracy of sinusoidal structures created using WaveShape can be increased by reducing the asymmetry. To reduce asymmetry, two adaptions were investigated. First a compensation term calculated from the difference profile was added to the laser power modulation used for structuring. With this adaption the shape deviation of an asymmetrical structure could be decreased by 66 % to a shape deviation of *Pa(Δ)* = 0.041. This result shows that asymmetry can be prevented using this adaption as the shape deviation is comparable to the value calculated for the symmetrical structure. With this adaption, asymmetry can be reduced without having to perform an extensive process parameter study. However, the difference profile is required which must be determined from a preliminary process step.

To reduce asymmetry in one process step, a modulation of the scanning speed during the process is investigated. Shape deviation can be decreased by 40 % to *Pa(Δ)* = 0.074. Thus, asymmetry is not as effectively prevented as when using a laser power modulation with a compensation term. However, the adaption can be performed without an extensive process parameter study and the difference profile is not required.

Another aim was to investigate destructuring. With conventional destructuring using the inverse laser power modulation the structure height of the symmetrical structure could be reduced by 83 % while the structure height of the asymmetric structure could only be reduced by 68 % to *Pa* = 3.54 μm.

To increase the efficiency of destructuring asymmetric structures, iterative destructing is investigated. With two iterations of destructuring, the structure height was reduced by 90 % to *Pa* = 0.967 μm. The reduction is larger than the reduction for destructuring symmetric structures.

As a second approach for more efficient destructuring of asymmetric structures an adaption of the laser power modulation via a compensation term is investigated. The structure height could be reduced by 86 % to *Pa* = 1.48 μm. The reduction of the structure height is thus larger than for conventional destructuring with inverse laser power modulation, but smaller in comparison to iterative destructuring.

## Data availability statement

Data will be made available on request.

## CRediT authorship contribution statement

**Laura Kreinest:** Writing - original draft, Methodology, Investigation, Data curation, Conceptualization. **Benedikt Schober:** Methodology, Investigation, Data curation. **Edgar Willenborg:** Writing - review & editing, Conceptualization. **Jochen Stollenwerk:** Writing - review & editing, Conceptualization.

## Declaration of competing interest

The authors declare that they have no known competing financial interests or personal relationships that could have appeared to influence the work reported in this paper.
